# Recent advances and potential applications of cross-kingdom movement of miRNAs in modulating plant’s disease response

**DOI:** 10.1080/15476286.2022.2062172

**Published:** 2022-04-20

**Authors:** Tilahun Rabuma, Om Prakash Gupta, Vinod Chhokar

**Affiliations:** a Department of Bio and Nano Technology, Guru Jambheshwar University of Science and Technology, Hisar, INDIA; b Department of Biotechnology, College of Natural and Computational Science, Wolkite University, Wolkite, Ethiopia; c Division of Quality and Basic Sciences, ICAR-Indian Institute of Wheat and Barley Research, Karnal, INDIA

**Keywords:** miRNAs, cross kingdom movement, spray-induced gene silencing (SIGS), crop protection, biotic stress

## Abstract

In the recent past, cross-kingdom movement of miRNAs, small (20–25 bases), and endogenous regulatory RNA molecules has emerged as one of the major research areas to understand the potential implications in modulating the plant’s biotic stress response. The current review discussed the recent developments in the mechanism of cross-kingdom movement (long and short distance) and critical cross-talk between host’s miRNAs in regulating gene function in bacteria, fungi, viruses, insects, and nematodes, and *vice-versa* during host-pathogen interaction and their potential implications in crop protection. Moreover, cross-kingdom movement during symbiotic interaction, the emerging role of plant’s miRNAs in modulating animal’s gene function, and feasibility of spray-induced gene silencing (SIGS) in combating biotic stresses in plants are also critically evaluated. The current review article analysed the horizontal transfer of miRNAs among plants, animals, and microbes that regulates gene expression in the host or pathogenic organisms, contributing to crop protection. Further, it highlighted the challenges and opportunities to harness the full potential of this emerging approach to mitigate biotic stress efficiently.

## 1. Introduction

MicroRNAs (miRNAs) are endogenous, small non-coding RNA molecules with sizes ranging from 20 to 25 bases [1,2], which negatively regulate gene expression at the post-transcriptional level [3]. They are one of the most abundant classes of gene regulatory molecules, regulating the expression of many growths and development associated protein-coding genes during the entire cycle of a multicellular organism [4]. MicroRNA was first discovered as *lin-4* in *Caenorhabditis elegans* (*C. elegans*) [5–7]. Since then, thousands of miRNAs have been identified in plants, animals, and other eukaryotic organisms [8]. In plants, miRNAs were first discovered in *Arabidopsis thaliana* and subsequently in other plant species [9,10]. The latest release of miRbase (v22) was reported to contain 38,589 hairpin precursors and 48,860 mature microRNAs sequences from 271 organisms showing a continuous increase in the miRNA pool [11,12]. So far, about 8433 miRNAs from 121 plant species have been archived in the plant miRNA database (miRBase) [13]. Moreover, 16,422 novel miRNAs from 88 plant species were archived in the plant miRNA Encyclopaedia (PmiREN, http://www.pmiren.com/) [14]. The PmiREN v.2.0 latest release contains 38,186 known miRNAs belonging to 7,838 families with a predicted 141, 327 miRNA-targets pairs in 179 plant species [15]. These miRNAs can control a broad range of biological processes by modulating their corresponding target genes expression [16,17], involved in a vast range of plant functions, including leaf morphogenesis [18], root development [19,20], growth transition [21], reproductive stage [22], disease resistance [23,24], *etc.*

The miRNAs involved in modulating diseases response regulate their target gene expression either through up or down-regulation upon fungal infection [25,26]. For instance, Gupta et al. [26] reported a significant accumulation of *miR1138* in bread wheat infected with *P. graminis f.sp. tritici* (62G29-1). The earlier speculation supports the idea of miRNAs targeting the pathogen’s genes in the host cell upon infection, and to counter the host defence, the pathogen’s small RNA mediates the targeting of host defence-related genes. The miRNAs targeting pathogen’s genes can be achieved by the cross-kingdom transfer of small RNAs from the host to the pathogens. The first report of cross-kingdom transfer of small RNA from host to pathogen and vice-versa in *Botrytis cinerea*-Arabidopsis and *Lycopersicon esculentum* pathosystem [27] has unlocked a new area on small RNA-based plant-pathogen interaction for further exploration. This, during the last decade, enabled extensive work on cross-kingdom systemic, *i.e*. host (plant & animal) to the pathogen (bacteria, fungi, viruses, insects, etc.) and vice-versa, movement of small RNA [28]. Moreover, with rapid advancement in molecular understanding, the research area on the potential applications of cross-kingdom movement of small RNAs in crop protection is gaining more familiarity [29,30]. Considering the quantum of information coming daily on the cross-kingdom movement of small RNAs, we synthesized this review to critically evaluate the existing trends, challenges and opportunities in utilizing this approach in crop protection against biotic stresses.

## 2. Biogenesis of miRNAs: miRNA transcription and maturation

**Figure 1.**
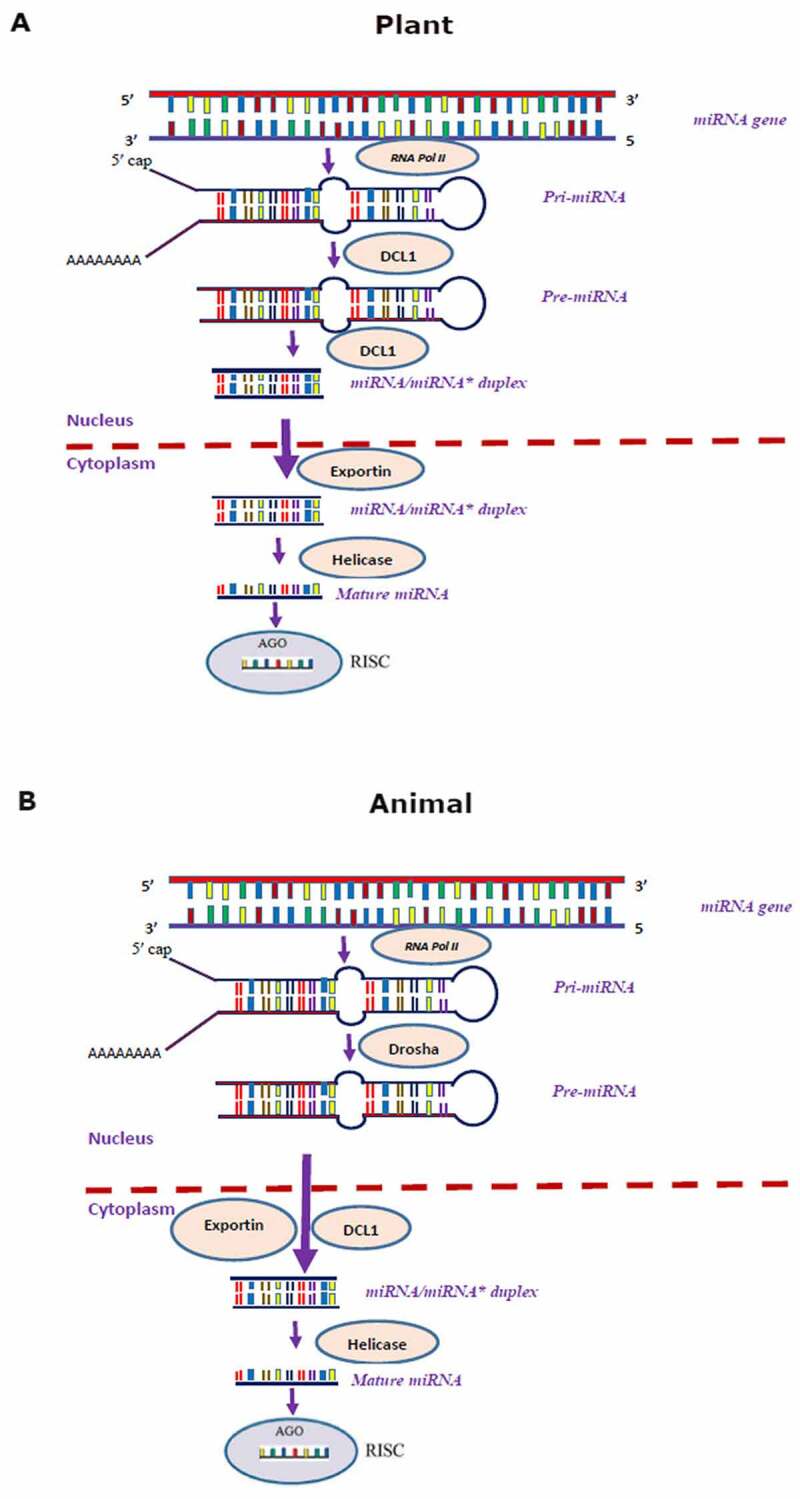
Biogenesis pathways of miRNAs in (A) plants; and (B) animals.

miRNA sequence specificity with its corresponding target gene is necessary for regulating their expression in both plants and animals [20]. Earlier reports suggest that most animal and plant miRNAs regulate the expression of their corresponding target genes by triggering translational repression and mRNA cleavage, respectively [21,22]. In contrast, few reports suggest miRNA-mediated translational inhibition in plants [23,24]. Despite the cohesion in the mode of action of miRNAs in plants and animals, there are significant differences in their biogenesis [8,31]. The loci that produce miRNAs have distinct genomic arrangements in each kingdom, and miRNAs are excised from precursor transcripts by different pathways in the two kingdoms [8]. The biogenesis pathway of miRNA in both plants and animals is depicted in Fig. 1. The miRNAs are primarily synthesized as primary transcripts (pri-miRNA) with 5ʹ capping and polyadenylation at 3ʹ end by RNA polymerase II and III in plants and animals [32]. In-plant cells, the pri-miRNAs are processed using Dicer-like 1 protein (DCL 1) to remove poly-A tail generating pre-miRNAs [33–36]. The looped secondary structure of pre-miRNAs are further processed by DCL 1, resulting in miRNA-miRNA* (guide-passenger strand) duplexes [37], which is transported from the nucleus to cytoplasm with the help of exportin transporter [38]. Finally, the duplex gets separated in the cytoplasm, and matured strand of miRNA is incorporated with an RNA-induced silencing complex (RISC) that acts as a guide for mature miRNA to recognize the complementary site of its target gene [32].

In animals, the pri-miRNAs poly-A tails are removed with the help of a microprocessor complex, minimally composed of Drosha, an RNase III enzyme, resulting in pre-miRNA hairpin [39,40], with a 5’ monophosphate group and a 2-nt 3’-end overhang [40]. The pre-miRNAs are simultaneously processed and exported from the nuclease to the cytoplasm with the help of DCL 1 and exportin-5 (XPO5) in the presence of its Ran-GTP co-factor, forming miRNA/miRNA* duplex [41–43]. Once in the cytoplasm, GTP hydrolysis resulted in the dissociation of pre-miRNA from XPO5 [40]. The RNA poly III enzymes cleave the pre-miRNA hairpin loops to produce a ~ 22 bp mature miRNA duplex [44,45]. Then, the mature miRNA is formed by helicase. Finally, the RNA binding proteins and PACT (protein activator of PKR) associate with Dicer *in vivo*, facilitating the assembly of matured miRNA into RISC to perform its regulatory function [46,47].

A difference in the location of the binding site of miRNA within the target region between animals and plants was detected. For instance, in animals, the binding site usually occurs in multiples and always within the 3′ untranslated region (3′-UTR) of the mRNA, while plant miRNA-binding sites are found almost exclusively within the open reading frames (ORF) of the target genes [48]. However, in few plants, the binding site of miRNAs is predicted to occur in 3′-UTR of mRNA [49]. Hence, the number of miRNA binding sites and their location reflect a significant mechanistic difference between animals and plants [48]. Even though there are several differences in miRNAs binding sites between animals and plants, in both kingdoms, miRNAs regulate target gene expression either by inhibiting translation through a slicer-independent mechanism [50] or negatively controlling the protein-coding sequence via mRNA-directed cleavage mechanism at a post-transcriptional level [26,28,51]. Moreover, in both plants and animals, miRNAs sequence specificity with their corresponding target is necessary to regulate gene expression [20], determining whether the target gene is cleaved or translationally inhibited [9,22].

## 3. Disease pathogenesis and plant defence modulated by miRNAs

Plants are often more prone to different biotic and abiotic stresses owing to their sessile nature, and constant exposure to an unpredictable environment leading to extreme loss to crop productivity [52]. The overexpression, up- or down-regulation, or knock-in of transcribed miRNA gene sequences has confirmed the involvement of miRNAs in biotic stress responses in different plant species [53]. For instance, overexpression of *miR396* in rice leads to an enhanced susceptibility to *M. oryzae* [54], whereas overexpression of *miR164* and miR396 significantly improved tolerance to cyst nematode [53]. Furthermore, the overexpression of miR827 increased susceptibility to *H. schachtii*, whereas the expression of a miR827-resistant *NLA* decreased plant susceptibility [55]. An induced expression of miR166 under *Rhizoctonia solan*i infection in susceptible and resistant rice cultivars suggest basal response regulators [56]. Similarly, an increase in the accumulation of miR166 and miR159 in cotton plants in response to fungal pathogen *Verticillium dahliae* infection was reported [57]. Overexpression of *miR393* represses auxin signalling, enhancing bacterial resistance, suggesting auxin signalling plays a vital role in plant-induced immune response [58,59]. The complementary strand miR393 has also been reported to play a role in antibacterial immunity by negatively regulating the expression of MEMB12 (SNARE), a protein involved in membrane fusion, thereby promoting the exocytosis of pathogenesis-related protein (PR1) [52]. Natarajan et al. [60] demonstrated that *miR160* plays a crucial role in local defence and systemic acquired resistance (SAR) responses by regulating targets of auxin response factor (*StARF10)* and MAP kinase (*StMAPK9*) during the interaction between potato and *P. infestans*. Moreover, *miR160a* positively regulates PAMP-induced callose deposition, whereas miR398b and miR773 negatively regulate PAMP-induced callose deposition and disease resistance to bacteria, suggesting a complexity of the miRNA regulation in plant innate immunity [61]. Hence, miRNAs have been shown to modulate plant defence responses at various levels as regulation of gene expression by miRNAs is a crucial mechanism in facilitating the response of plants against biotic stress [62]. Despite this advancement, further targeted work on functional validation of the role of miRNA in regulating the expression of genes utilizing emerging reverse genetic technologies such as CRISPR/Cas 9 technology is critically required to broaden the current horizon of miRNA-target gene-mediated disease cross talk.

## 4. Advances in cross-kingdom movement and role of host miRNAs during host-pathogen interaction

**Figure 2.**
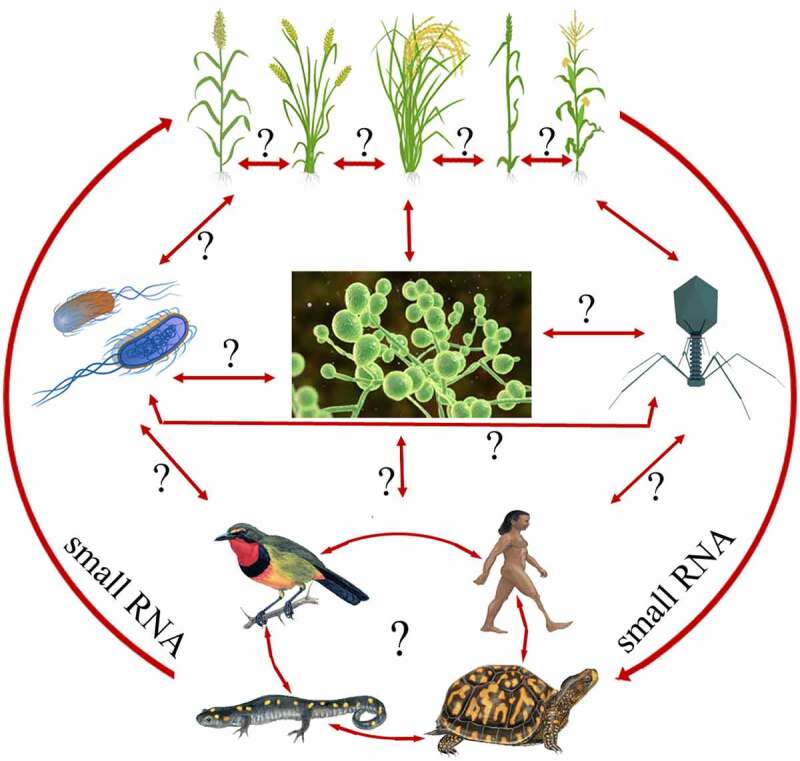
A hypothetical model representing the cross-kingdom movement of sRNA. Question mark (?) represent the unavailability of information in literatures.

In the recent past, several reports believe that movement of sRNA, especially miRNAs have no boundary, *i.e*. they can move not only within cells/tissues within the individual organism but also across the kingdom in different eukaryotic species or species to species [28,63–67]. This type of signal transfer across the kingdom between distantly related species is termed cross-kingdom RNA interference (RNAi). Fig. 2 represents the hypothesis of all possible interactions for the cross-kingdom movement of small RNA. Micro RNAs have been reported for their potential transfer to distantly related organisms, where they exert a regulatory role in cross-kingdom fashion [68]. The conserved features of the RNA silencing machinery among eukaryotes favour cross-kingdom miRNA transfer, though taxon-specific variations exist [68]. Such type of variation is mainly related to the ability of organisms to incorporate RNA molecules into other tissues/cells, silencing the target gene expression [69,70].

The cross-kingdom miRNA transfer has been observed in host-pathogen relations, inhibiting invasive pathogen powers [68]. Plants are attacked by a large number of pathogens such as bacteria, fungi, mycoplasma, nematodes, viruses, viroids, and parasites, and they have developed a defence strategy against these pathogens [71,72]. Due to evolved nature of plants, they have developed a sophisticated mechanism of resistance against pathogens through miRNA-guided transcriptional or post-transcriptional silencing of pathogenic mRNA of virulence genes. Growing reports have sufficiently demonstrated the potential implications of many plant’s miRNAs in defence response against various pathogens [32,72], see review [73]. To enumerate a few, resistance mechanism in cotton plants against fungal pathogen has been demonstrated by miRNA-based targeting of virulence gene [74]. The *miR1138* was highly accumulated in wheat infected with *P. graminis f.sp. tritici* (62G29-1) [26]. Similarly, Yin et al. [75] have reported the potential role of cotton miRNAs enhancing resistance against *Verticillium dahlia* infection. Overexpression of *miR160a* and *miR398b* in transgenic rice displayed enhanced resistance against *Magnaporthe oryzae* infection, resulting in decreased fungal growth and up-regulation of defence-related genes [76]. Antibacterial immunity was activated by *miRNA393*-AGO1 mediated suppression of auxin receptors [77]. Recently, Kuntala and Niraj [78] have reviewed the status of miRNAs’ role in plant-insect interactions. Moreover, the cross-kingdom plant-derived *miR159a, miR166a-3p*, and the *novel-7703-5p* were demonstrated to influence cellular and metabolic processes in *P. xylostella* [68,79]. Despite significant efforts that have been made in deciphering the role of host miRNA during host-pathogen interaction along with cross-boundary movement, further comprehensive work involving several hosts and pathogens could be useful in reorienting our current understanding. This understanding will help molecular breeders and pathologists devise a suitable strategy to mitigate pathogen infestations.

## 5. Promising mechanism of long and short distance cross-kingdom movement of miRNAs

Plant-derived miRNAs can be transferred to the animal via diet/plant vegetables [80]. Diet/plant-derived miRNAs were reported in the serum of human/plant-feeding animals, regulating gene expression in recipients in a sequence-specific manner [3]. Plant miRNAs can act as a bioactive constituent of the plant, which has the potential of travelling from plants to animals via the gastrointestinal (GI) tract to access its target, modulating gene expression in the recipients [81]. It is proposed that diet/plant-derived miRNAs are absorbed by the intestinal epithelial cell and packaged into microvesicles (MVs) to shelter degradation and subsequently released into blood circulation [81]. The miRNAs are then distributed to various tissues/cells, where they perform regulation of target gene expression [3]. Plant-derived miRNAs can also be associated with animal AGO2 protein forming RNA-induced silencing complex (RISC) to perform their function in the animal system [3]. Small RNAs can move locally between cells through plasmodesmata and over long distances through phloem [82]. In addition, sRNA can also move *via* symplast and apoplast in the plant.

During the long-distance travel of plant miRNAs to animals, questions arise about how they can survive in the animal’s gastrointestinal tract (GI), enter the blood circulatory system, and eventually identify their potential target genes [81]. For degradation resisting in the animal’s gut, the 3′-terminal nucleotide of plant’s miRNAs is 2’-O-methylated, enhancing the stability of miRNAs to ensure their regulatory function in animals [81,83]. Most plant miRNAs displayed modest resistance in the acidic gastric environment of animals [84]. The increased stability in an animal might also be ensured by the high GC content of plant-derived miRNAs [81]. For instance, a high GC content of *MIR2911* may increase its digestive stability [85,86]. Most importantly, the carriers of plant-derived miRNAs are more likely to protect the miRNAs from enormously punitive surroundings and support their movement into mammals [87]. Moreover, plant-derived miRNAs can be orally administered to animals for the treatment for therapeutic application. For instance, oral administration of miR159 mimic significantly suppressed the xenograft breast tumours in mice [88].

**Figure 3.**
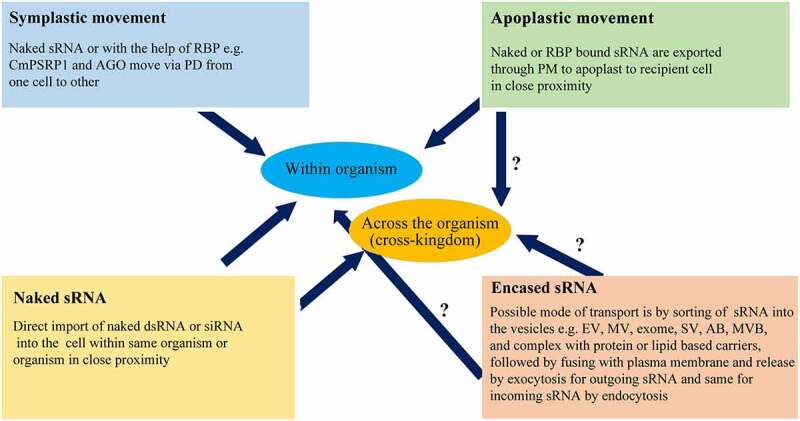
Description of strategies of sRNA movement across the species.

Wang et al. [80] have analysed two different mechanisms by which endogenous miRNAs can be incorporated into distantly related species, *i.e*. the use of the systemic RNA interference deficient (SID) transmembrane channel-mediated proteins and microvesicle (MV) compartments. Moreover, evidence supported that the sRNA is transferred either as a naked molecule or mediated by vesicles encasing. Different strategies utilized for sRNA movement have been described in Fig. 3. For instance, in and between plants and fungi, the sRNA can be transported through naked form, combined with RNA-binding proteins, or enclosed by vesicles [89]. In trans-kingdom transportation of small RNAs between plant and fungi, small RNAs inside vesicles can be transported from cell to cell through plasmodesmata (PM) which secreted through the plant plasma membrane (PPM) and then plant cell wall (PCW) to extracellular spaces, where they can also be taken up by fungal cell through fungal cell wall [89]. This transportation of sRNAs can be bidirectional, *i.e*. the small RNAs can be transferred through the fungal plasma membrane (FPM)–fungal cell wall (FCW)–extra‐invasive hyphae matrix (EIHMx)‐extra‐invasive hyphae membrane (EIHM) and then to plant cytoplasm pathway [89]. Even though different strategies for the cross-kingdom movements of miRNA were explained, the mechanism by which the exogenous miRNAs are loaded onto Argonaute proteins of distantly related species to produce a functional miRNA form has still needs to be explored in detail [80]. Therefore, the fungal cell wall plays an indispensable role in controlling sRNA movement between host and fungal cells.

## 6. Role of host miRNA in regulating the pathogen’s gene expression

MicroRNAs play an essential role in regulating the host’s biological, biochemical and physiological pathways against pathogen (viruses, fungi, parasite, and bacterial) infection by modulating the gene expression and deviation in cellular alignments [90]. The host’s RNAi silencing machinery has the potential capacity to directly target the RNA genome and related transcripts of several pathogens such as viruses, virus satellites, and viroids, to regulate the transcripts accumulation [91]. This silencing is performed by exporting specific plant sRNAs, including miRNAs, to induce cross-kingdom gene silencing in pathogenic fungi, thereby conferring disease resistance [30,74]. For example, siRNAs enter Oomycete *Phytophthora* via extracellular vesicles, silencing *Phytophthora* virulence genes to confer resistance in *Arabidopsis* during infection [30]. Similarly, *Arabidopsis miR166* was exported to *V. dahlia* fungal hyphae to suppress pathogenicity [92]. A comprehensive list of sRNA moving from plants to the pathogen is given in Table 1. Moreover, Zhang et al [74]. have investigated the transfer of the two miRNAs (*i.e. miR159* and *miR166*) from the cotton plant into *Verticillium dahliae* hyphae after infection. These two miRNAs have targeted the expression of the *Verticillium* genes coding for Ca^2+^-dependent cysteine protease (Clp-1) and isotrichodermin C-15 hydroxylase (HiC-15), respectively, associated with triggering fungal virulence [74]. Tinoco and co-workers reported translocation of silencing signals across the germinated spores from transgenic tobacco into *F. verticillioides* cells [93].

**Table 1.** List of sRNAs moving from plants to pathogens

Plants	Pathogen	miRNA	Target Gene	Function	Reference
**Fungi**					
Cotton	*V. dahliae*	miR159 and miR166	VDAG_09736 gene (encodes the Ca2+-dependent cysteine protease calpain clp-1)	Suppresses Clp-1 mRNA accumulation in *V. dahliae* hyphae in infected cotton plants to direct gene silencing in a fungal pathogen.	[74]
Arabidopsis	*V. dahliae*	miR166	Virulence genes	Silence virulence-related genes	[92]
Wheat	*F. graminearum*	RNAi	β-1,3-glucan synthase gene *FcGls1*	Silencing constructs revealed aberrant, swollen fungal hyphae, indicating severe hyphal cell wall defects	[125]
Transgenic tobacco with GUS-RNAi	*Fusarium verticillioides*	siRNAs (RNAi)	*gus* transgene	*GUS* gene silencing in transformed *Fusarium verticillioides*	[93]
Transgenic lettuce	*Bremia lactucae*	siRNAs	Highly Abundant Message #34 (HAM34) or Cellulose Synthase (CES1) genes of B. lactucae	Specifically suppressed expression of these genes, resulting in greatly reduced growth and inhibition of sporulation of B. lactucae.	[126]
Tomato and Arabidopsis	*V. dahliae*	RNAi	Genes encoding Ave1, Sge1 and NLP1	Targeting gene encoding Ave1, Sge1 and NLP1 leading suppression of Verticillium wilt disease on treatment	[127]
Transgenic Tobacco	*Sclerotinia sclerotiorum*	siRNA	Chitin synthase(chs) gene	Silencing of the fungal *chs* gene	[128]
wheat	*F. culmorum*	RNAi	β-1, 3-glucan synthase gene FcGls1	Caused a reduction of corresponding transcript levels in the pathogen and reduced disease symptoms.	[125]
Transgenic potato	*P. infestans*	RNAi	PiGPB1 gene	Targeted the G protein β-subunit (PiGPB1) important for pathogenicity resulted in most restricted disease progress.	[129]
Tall fescue	*Rhizoctonia solani*	RNAi	Essential genes(genes encoding RNA polymerase, importin beta-1 subunit, Cohesin complex subunit Psm1, and a ubiquitin E3 ligase) from R. solani	Suppress expression of genes (encoding RNA polymerase, importin beta-1 subunit, Cohesin complex subunit Psm1, and a ubiquitin E3 ligase) inside the fungus and thus inhibit fungal infection.	[130]
Barley	*Blumeria graminis*	RNAi	Effector gene *Avra10*	Resulted in reduced fungal development	[131]
Wheat	*Puccinia striiformis* f. sp. *tritici*	RNAinterference (RNAi)	Calcineurin homologs *Pscna1/Pscnb1*	Slowerextension of fungal hyphae and reduced production of urediospores	[132]
Wheat	*P. striiformis* f. sp. *tritici*	RNA interference (RNAi)	MAPK kinase gene *PsFUZ7*	Hyphal development strongly restricted, necrosis of plant cells in resistance responses induced	[133]
Wheat	*Puccinia striiformis* f. sp. tritici (Pst)	siRNAs	PsCPK1 gene(a PKA catalytic subunit gene)	Significant reduction in the length of infection hyphae and disease phenotype	[134]
Arabidopsis and tomato	*V. dahliae*	RNA interference (RNAi)	Three previously identified virulence genes of *V*. *dahliae* (*Ave1, Sge1*, and *NLP1*)	Reduced verticillium wilt disease in two of the three targets	[127]
Transgenic *S. tuberosum*	*P. infestans*	amiRNAs	Avr3a	Fungal virulence	[135]
Arabidopsis	*B. cinerea*	miR173	Target mRNAs	Silencing of pathogen virulence	[92]
Arabidopsis	*Phytophthora*	siRNAs	Target suppressors of RNAi (PSRs) gene	*Phytophthora* infection increases production of a pool of secondary siRNAs in *Arabidopsis* leads to developmental deficiency and abolishes virulence	[62,136]
**Virus**					
*L. japonica*	Influenza A viruses (IAVs)	miR2911	PB2 and NS1	Inhibited H1N1-encoded PB2 and NS1 protein expression	[85]
**Insects**					
Arabidopsis	*P. xylostella*	miR159c	BJHSP1	Pupae development	[79]
Transgenic Tobacco	*M. persicae*	amiRNAs	MpAChE2	Synaptic transmission	[137]
Transgenic Arabidopsis	*H. armigera*	amiRNAs	HaAce1	Synaptic transmission	[138]
Transgenic Tobacco	*H. armigera*	amiRNAs	Chitinase	Chitin synthesis	[139]
Transgenic Rice	*C. suppressalis*	amiRNAs	CsSpo CsEcR	Embryonic development	[140]
B. campestris	*A. mellifera*	miR162a	amTOR	Delay development and decrease body and ovary size in honeybee and regulate caste development at larval stage	[110]
S. bicolour	*Schizaphis graminum*	sbi-miR5163-3p	Butanoate (Butyrate) metabolism gene	Supress Butanoate (Butyrate) metabolism gene expression	[101]
H.vulgare	*Sipha flava*	sbi-miR2-3p	Drug metabolism-P450 and Metabolism of xenobiotics by P450 (00980*)	Supress gene of drug metabolism gene	[101]

Zhu and co-workers reported that compared to royal jelly, beebread harbour more plant miRNAs that decrease ovary and body size in honeybees. This hinders the differentiation of larvae into queens leading to more worker bees [70]. Plant-parasitic nematodes are responsible for considerable crop losses worldwide [68]. The most scientific literature on gene silencing mechanisms comes from nematodes, specifically from *Caenorabditis elegans* [68]. However, most of these studies emphasize on uptake of dsRNAs from the surroundings than on the cross-kingdom movement of plant miRNAs [68,94,95]. Over the years, significant progress has been made in deciphering the role of plant miRNAs against phytonematodes infection [68,96–99]. Zhang and co-workers observed that miR166a-3p, miR159a, and the novel-7703-5p target *BJHSP2, BJHSP1 (*(basic juvenile hormone-suppressible protein 1 and 2) and *PPO2* (polyphenol oxidase subunit 2) genes which affects metabolic and cellular processes in *P. xylostella* [53]. For instance, Zhang and co-workers confirmed a modest level of plant-derived *miR168* in *Lepidoptera* and *Coleoptera* species [100]. Wang and co-workers predicted 13 sorghums (*Sorghum bicolour*) miRNAs and three barley miRNAs in Aphid targeting aphid genes playing essential roles in sucrose and starch metabolism and detoxification [101]. Despite this, the precise role of exogenous plant miRNAs on herbivore gene expression still needs to be functionally elucidated.

## 7. Evidence and advances on the role of pathogen’s miRNA in modulating the host gene expression

The evidence-based science of cross-kingdom movement of sRNA has recently gained significant attention, with a plethora of research being performed in different hosts and pathogens. Available reports suggested that sRNAs derived from pathogens can also work as an effector molecule and modulate host gene expression as a counter defence strategy. For instance, The novel miRNA (Pst-milR1) in *Puccinia striiformisf. sp.tritici* takes part in cross-kingdom RNA interference (RNAi) events by binding the pathogenesis-related 2 (PR2) (b-1,3-glucanase SM638) gene in wheat [102] that might suppress the host-mediated defence strategy in its counter defence. Similarly, Bc-sRNAs derived from *Botrytis cinerea* binds with Argonaute 1 (AGO1) and capture the host RNAi machinery leading to selective silencing of host immunity genes [27], suggesting that the *B. cinerea* transfers virulent sRNA effector molecules into host plant cells to suppress host immunity as a counter defence strategy to achieve infection [27]. Wang and co-workers functionally validated the role of Bc-siR37 as an effector molecule that is predicted to target several *Arabidopsis* genes associated with disease pathogenesis, such as receptor-like kinases, WRKY transcription factors, and cell wall-modifying enzymes upon *B. cinerea* infection [103] Brilli et al. [104] identified bidirectional interaction between pathogen-host, *i.e*. the sRNA produced by *Plasmopara viticola* triggered the cleavage of grapevine (*Vitis vinifera*) genes, while the sRNAs produced from grapevine target the *P.viticola* mRNAs. An updated list of sRNA moving from pathogen to plants and their regulatory roles has been given in Table 2.

**Table 2.** List of sRNAs that move from pathogens to plants

Plants	Pathogen	miRNA	Target Gene	Function	Reference
**Fungi**					
*A. thaliana*	*Botrytis cinerea*	Bc-siR3.2	Mitogen-activated protein kinases (MAPK2 and MAPK1)	Suppress mitogen-activated protein kinases (MPK2 and MPK1) function in plant immunity	[27]
*V. vinifera*	*Plasmopara viticola*	sRNAs	Immunity gene	mRNAs for cleavaged during suppressing plant immunity	[104]
*A. thaliana*	*Botrytis cinerea*	Bc-siR3.1	PRXIIF	Suppress Peroxiredoxins gene	[27]
*S. lycopersicum*	*Botrytis cinerea*	Bc-siR3.2	MAPKKK4	*MAPKKK4* was suppressed upon *B. cinerea* infection	[27]
*Arabidopsis thaliana*	*Botrytis cinerea*	Bc-siR37	At-*WRKY7*, At-*PMR6*, At-*FEI2*	*Suppress* At-*WRKY7*, At-*PMR6*, At-*FEI2 genes* which encode an immune-related transcription factor, a pectin lyase, and a leucine-rich repeat (LRR) receptor kinase, respectively	[103]
*T. aestivum*	*P. striiformis*	pst-milR1	SM638	Innate immunity	[102]
*T. aestivum*	*P. striiformis* f. sp. *tritici*	pst-milR1	Wheat pathogenesis-related 2 (PR2) gene	By binding the wheat pathogenesis-related 2 (PR2) gene (represses the plant immune response by suppressing the expression of PR2 increased the susceptibility of wheat to the aviru-	[102]
**Virus**					
*V. vinifera*	*Grapevine fleck virus* (GFkV)	vsiR1378	S2P metalloprotease gene	targets the transcript TC107032 coding for a putative S2P metalloprotease	[141]
*V. vinifera*	*Grapevine rupestris stem pitting-associated virus* (GRSPaV)	vsiR6978	Vacuolar protein-sorting 55 (VPS55)	transcripts (TC109537) coding for a vacuolar protein-sorting 55 (VPS55)	[141]
*Arabidopsis* *thaliana*	Cucumber mosaic virus	siRNAs	DCL2–DCL4 and RDR1 and RDR6	Induces more severe disease symptoms	[142]
*A. thaliana*	*Tobacco rattle virus*	siRNAs	DCL2–DCL4 and RDR1, RDR2 and RDR6	Induces more severe disease symptoms	[143]
**Parasitic plant**					
Dodders (*Cuscuta campestris. Cuscuta* spp.	*Arabidopsis thaliana*	Short interfering RNA (siRNA)	Target gene silencing (target host messenger RNAs)	Resulting in mRNA cleavage, secondary siRNA production, and decreased during parasitism mRNA accumulation and these trans-species miRNAs from *C. campestris* function to silence host genes in order to increase parasite growth and fitness	[144,145]

In addition to the pathogens’ miRNAs modulating host defence response, various molecules or effectors from pathogen reported to interfere with the host defence mechanism during pathogen interaction. Interestingly, plant viruses encode viral suppressors of RNA silencing (VSRs) molecule, interfering with host RNA silencing through multiple modes of action [105,106]. The plant virus-encoded VSR physically interacts with AGO1 to prevent miRNA or siRNA loading or degrading AGO1 protein [14,107]. For instance, the tombusvirus P19 protein (a type of VSRs) binds and sequesters plant miRNAs to suppress their activity in AGO, resulting in the increased loading of miR168 into AGO1 and subsequently reduced accumulations of cellular AGO1 [108,109]. Further research in the area of comprehensive characterization of pathogen’s miRNAs and their functional validation in several models and non-model plants would broaden our current understating, which will guide us in devising suitable mitigation strategies against pathogen mediated crop losses.

## 8. Cross-kingdom movement of miRNAs during symbiotic interaction

Small RNA-based cell-to-cell communication occurs between an organism of different species by transporting regulatory molecules across the cellular boundaries between the host and its interacting pathogens/symbionts [67]. The cross-kingdom transfer of miRNAs between symbiotic or mutualistic relations impacts mutualistic relations and the performance of different agricultural crop plants [68]. The miRNAs cross-transferred from the plant through symbiotic/mutualist relation reported influencing the growth and developmental stage of the receiving organisms [110]. The Arbuscular Mycorrhizal Fungi (AMF) is an important component of the host plant’s root providing several benefits, including improving nutrient uptake and tolerance to various stress. Even though little is now about RNAi mechanism and sRNAs occurrence in Arbuscular Mycorrhizal Fungi (AMF), several fungal sRNAs have the potential to target transcripts, including some specific mRNA in *Medicago truncatula* roots upon Arbuscular Mycorrhizal Fungi (AMF colonization [111]. The transfer of fungal sRNAs in symbiosis interaction modulates plant metabolic pathways and defence response [111]. Hence, the fungal sRNAs positively affect the symbiotic interaction between fungi and their host plant.

Moreover, in the mutualistic relation of plant-pollinator, the dietary intake of the plant miR162a was shown to regulate caste development at the larval stage of honey [68,110]. Hence, silencing TOR (target of rapamycin) by plant-derived miR162a blocks queen fate and results in individuals with worker morphology. A contrary report on the uptake of plant-derived miRNAs by recipient organisms has been observed. Snow et al. [112] observed negligible delivery of plant-derived miRNAs in recipient honeybees despite oral uptake of pollen containing these molecules, suggesting that the horizontal delivery of plant-derived miRNAs via dietary ingestion was neither a robust nor a frequent mechanism to maintain steady-state microRNA levels in receiving organisms. However, Masood et al. [113] revealed an accumulation of plant miRNAs after pollen ingestion in adult bees’ midguts without evidencing their biological role. They supported the premise that pollen miRNAs ingested as part of a typical diet were not robustly transferred across barrier epithelia of adult honey bees under normal conditions. The reports signifying cross transfer and accumulation of miRNA involved in the symbiotic relationship of plants and other organisms are limited. Moreover, more specialized or specific delivery mechanisms for more efficient cross-transfer of miRNAs between symbiotic/mutualistic relations will be required to be explored.

Contrary to the transferred role of sRNAs between plants and symbiotic/mutualistic organisms, the cross transfer of miRNAs from the plant to pathogen/parasitic or vice versa has a negative impact on the host or pathogen. For instance, the novel miRNA like RNA from *Puccinia striiformis* f. sp. *tritici* (*Pst*) to wheat suppressed its innate immunity [102]. This part was more discussed in section 7 above.

## 9. Application of cross-kingdom miRNA movement in crop protection

The movement of miRNAs across different species has various applications in crop protection in an environment-friendly manner. For instance, the *miRNA159* and *miRNA166* constitute an example of plant miRNA transfer to pathogenic fungi from cotton (*Gossypium hirsutum*), which confer resistance to *Verticillium dahlia* [74]. Hence, horizontal transfer of miRNA among plants, animals, and microbes regulates gene expression in the host or pathogenic organisms, contributing to crop protection that could efficiently be utilized in the breeding programme. The transfer of miRNAs from pathogens to hosts primarily involves suppressing plant defence mechanisms as a counter defence mechanism. Wang and co-workers showed that expressing sRNAs targeting Bc-DCL1 and Bc-DCL2 in *Arabidopsis* and tomato silences Bc-DCL genes and attenuates fungal pathogenicity and growth, exemplifying bidirectional cross-kingdom RNAi and sRNA trafficking between plants and fungi [70]. This indicates that the cross-kingdom transfer of miRNAs suppresses the plant pathogen’s virulence and protects the crop plant. Furthermore, exogenous uptake from the environment was discovered in particular fungal pathogens, suppressing the virulence capability of the related pathogen [114]. *Botrytis cinerea*, causing grey mould disease, has been taken external sRNAs and dsRNA through spraying on the surface of the fruit, vegetables, and flowers and targeting the fungal pathogen gene against plant infection [114]. Moreover, the plant also transfers ds-siRNAs into coleopteran insects, silencing their transcription and suppressing their growth [63].

## 10. Potential application of spray induced gene silencing (SIGS) for combating insect pests in plants

At present, crop breeders depend almost entirely on fungicides to control disease, resulting in pesticide residues that often endanger human health and the environment [115]. Different resistant strains of fungi have been identified against every primary fungicide used in the agricultural production system [116]. Therefore, there is an urgent need to develop an eco-friendly and effective mechanism of agricultural crop protection from pathogen invasion. Modern agriculture is now on the verge of the third green revolution; the knowledge generated by reverse genetics in the functional characterization of genes could be harnessed in agricultural pest management [117]. RNA-based technologies, especially RNAi, have tremendous potential to be a practical approach for plant protection. RNAi has been explored as a strategy for pest control by expressing insect-targeted dsRNA in host plants to specifically block the expression of essential genes, resulting in insect mortality [118]. Among RNAi methods, SIGS has emerged as an innovative strategy for crop protection [119]. RNA sprays that result in target gene silencing have been observed with viruses [120] and fungi [121–123]. SIGS significantly simulates HIGS (Host-Induced Gene Silencing) without the need to develop stably transformed plants and has been demonstrated to be effective in the control of both *F. graminearum* and *Botrytis cinerea* [121]. The dsRNA/siRNA-based SIGS has attracted attention due to its feasibility and low cost compared to transgenic plants, and the technology demonstrates a potential paradigm shift in crop protection [117,119]. The dsRNA sprayed onto plant surface enters fungal cells by two possible pathways, *i.e*. RNA can be taken first by the plant cell and transferred into pathogenic fungi and/or directly taken by fungal cells [121]. These RNAs subsequently work in two ways: the RNAs taken up by plant cells induce the plant RNAi machinery, and then the RNAs taken up by the fungal cells induce the fungal RNAi machinery directly [119]. Koch and his co-worker demonstrated that barley SIGS conferred resistance against *F. graminearum* by silencing *CYP51* genes [119]. They also demonstrated that spraying the RNA fragments of jellyfish green fluorescent protein (GFP) on barley leaves effectively silenced GFP expression in a GFP-expressing *F. graminearum* strain, potentially targeting any essential genes in various interacting pathogens [121]. Moreover, Werner and co-workers also found that targeting *ARGONAUTE* and *DICER* genes of *F. graminearum* (*Fg*), the fungal RNAi machinery via SIGS could protect barley leaves from *Fg* infection [124]. Additionally, the dsRNA sprays can inhibit *Botrytis cinerea* and *Sclerotinia sclerotiorum* growth on *Brassica napus* [123]. The effectiveness of SIGS to protect pathogen invasion is dependent on the pathogen type to take up the naked miRNAs/sRNAs/RNAi. The pathogen’s RNA uptake efficiency can largely determine the success of SIGS for plant disease management, and therefore, establishing the effectiveness of SIGS across a wide range of pathogens is a critical next step in developing this technology.

## 11. Conclusion and prospects

Plant pathogens are continually affecting crop production throughout the world. Here, we analysed the existing cross-kingdom transfer of miRNAs during plant-animal and plant-pathogen interaction. However, there are also contradictory scenarios; plant miRNAs would not have passed through ingestion but could be mixed due to contamination during the sequencing of miRNAs. Recently, the role of miRNAs in regulating gene expression in host and pathogen have given a big concern for controlling pathogen in crop plants. Further investigation of the miRNA-mediated process in plant-pathogen interactions is needed to devise novel strategies for controlling pathogen infection in crop plants and improving crop productivity. MicroRNA-mediated gene silencing has vital significance in plant immunity. miRNAs-based SIGS techniques can be used as a mechanism of crop plant protection from pathogen invention. Moreover, miRNAs could be used to be very useful as biomarkers for disease resistance characteristics in breeding programme. Further exploration of cross-kingdom transfer of miRNAs would facilitate a more in-depth understanding of miRNAs in gene silencing in the host organism and trans regulation of a gene in host pathogens.

## Declarations

**Ethics approval and consent to participate**

This research did not involve the use of any animal or human data or tissue.
